# Structural Basis of a Histone H3 Lysine 4 Demethylase Required for Stem Elongation in Rice

**DOI:** 10.1371/journal.pgen.1003239

**Published:** 2013-01-24

**Authors:** Qingfeng Chen, Xiangsong Chen, Quan Wang, Faben Zhang, Zhiyong Lou, Qifa Zhang, Dao-Xiu Zhou

**Affiliations:** 1 National Key Laboratory of Crop Genetic Improvement, Huazhong Agricultural University, Wuhan, China; 2 Laboratory of Structural Biology, Tsinghua University, Beijing, China; 3 High-Throughput Molecular Drug Discovery Center, Tianjin International Joint Academy of Biotechnology and Medicine, Tianjin, China; 4 College of Life Science, Nankai University, Tianjin, China; 5 Institut de Biologie des Plantes, Université Paris–Sud 11, Orsay, France; The University of North Carolina at Chapel Hill, United States of America

## Abstract

Histone lysine methylation is an important epigenetic modification in regulating chromatin structure and gene expression. Histone H3 lysine 4 methylation (H3K4me), which can be in a mono-, di-, or trimethylated state, has been shown to play an important role in gene expression involved in plant developmental control and stress adaptation. However, the resetting mechanism of this epigenetic modification is not yet fully understood. In this work, we identified a JmjC domain-containing protein, JMJ703, as a histone lysine demethylase that specifically reverses all three forms of H3K4me in rice. Loss-of-function mutation of the gene affected stem elongation and plant growth, which may be related to increased expression of cytokinin oxidase genes in the mutant. Analysis of crystal structure of the catalytic core domain (c-JMJ703) of the protein revealed a general structural similarity with mammalian and yeast JMJD2 proteins that are H3K9 and H3K36 demethylases. However, several specific features were observed in the structure of c-JMJ703. Key residues that interact with cofactors Fe(II) and N-oxalylglycine and the methylated H3K4 substrate peptide were identified and were shown to be essential for the demethylase activity *in vivo*. Several key residues are specifically conserved in known H3K4 demethylases, suggesting that they may be involved in the specificity for H3K4 demethylation.

## Author Summary

Genomic DNA is associated with histone proteins to form the basic structure of chromatin. Lysine residues within the N-terminal end of histones H3 and H4 can be methylated, which may have a positive or a negative effect on the activity of associated DNA or genes, depending on the position of the lysines in the histones. Histone lysine methylation can be reversed by histone demethylases. However, it is not very clear how the specificity of histone demethylases to different histone lysines is determined. In this work we have identified a rice histone demethylase, namely JMJ703, which specifically demethylates methylated histone H3 lysine 4. We found that loss of the enzyme reduces cell division rate of the stem and the size of plant stature, indicating the importance of the protein in plant growth. The crystal structure of the catalytic domain of the protein shares a general similarity with that of mammalian and yeast proteins that demethylate methylated histone H3 lysine 9 and lysine 36, but displays several distinct structural features that are important for substrate and cofactor binding and enzymatic activity of the protein. We found that key amino acids involved in the specific structures are conserved within known H3 lysine 4 demethylases, which may be involved in the specificity to histone H3 lysine 4.

## Introduction

Histone methylation is an important epigenetic modification for chromatin structure, genome function, and gene expression in eukaryotic cells [1]. Histone methylation can be reversed by histone demethylases [1]. Lysine Specific Demethylase 1 (LSD1) is the first identified histone demethylase characterized as a member of the flavin-dependent amine oxidase family [2]. The second class of histone demethylases featured with the jumonji C (JmjC) domain has been shown to catalyze histone lysine demethylation through ferrous ion (Fe(II)) and α-ketoglutaric acid (α-KG)-dependent oxidative reactions [3].

Structurally related JmjC domain-containing proteins are classified into 7 subgroups based on phylogenetic analysis of members from yeast and animal cells [4]. Among them, the JmjC domain-containing histone demethylase 1 (JHDM1) subgroup has been demonstrated to reverse mono- and dimethylated histone H3 lysine 36 (H3K36me1/me2) [3]. The JmjC domain-containing histone demethylase 2 (JHDM2) subgroup has been identified to reverse dimethylated histone H3 lysine 9 (H3K9me2) [5], whereas the JMJD2 (also called JHDM3) group specifically reverses methylated H3K9 and/or H3K36 [6]. UTX/UTY is involved in the reversal of histone H3 lysine 27 methylation (H3K27me) [7], whereas PHF8 which belongs to PHF2/PHF8 subfamily demethylates H3K9me1/2, H3K27me2 and monomethylated histone H4 lysine 20 (H4K20me1) [8]–[10]. Aside from these members, the JARID subgroup is responsible for H3K4 demethylation [11]. The JmjC domain-only subgroup has the smallest molecular architecture; members of which have been shown to catalyze divergent reactions, such as histone arginine demethylation [12] and asparagine protein hydroxylation [13]. More recently, JMJD6 is shown to catalyze protein lysyl-hydroxylation [14].

Plant JmjC proteins have been shown to play important roles in the regulation of epigenetic processes, growth and development [15]. Although conserved with yeast and animal homologues, plant JmjC proteins display several distinct features. For instance, the UTX subgroup proteins that exhibit H3K27 demethylase activity are not found in plants. Recent data show that RELATIVE OF EARLY FLOWERING 6 (REF6), a member of the JMJD2 subgroup, can demethylate H3K27 in *Arabidopsis* [16]. Conversely, there exists a subgroup of plant JmjC proteins that include additional protein modules that are missing from animal or yeast homologues [17]. The amino acid sequence of the JmjC domains of this subgroup is closely related to JARID, while the overall domain organization of the core protein is similar to that of the JMJD2 group [17], [18]. To study this plant specific group of JmjC proteins, we analyzed the developmental function, enzymatic activity, and crystal structure of JMJ703, a member of this subgroup in rice. Our results demonstrate that JMJ703 is essential for plant cell division and stem elongation and specifically demethylates mono-, di-, and trimethylated H3K4 *in vivo* and *in vitro*. The high resolution structure of the catalytic core of JMJ703 (c-JMJ703) in complex with cofactors and substrate peptide reveals that the overall folding of c-JMJ703 is similar to those defined in animal and yeast JMJD2 that are H3K9 and H3K36 demethylases. However, the crystal structure of c-JMJ703 displays a number of specific features in cofactor interaction and in substrate peptide binding. Substitution mutation analysis indicated that residues implicated in the specific structures are essential for the enzymatic activity, some of which are conserved within JARID proteins and plant H3K4 demethylases and may be involved in the specificity of this class of enzymes.

## Results

### *JMJ703* is essential for rice stem elongation

**Figure 1:**
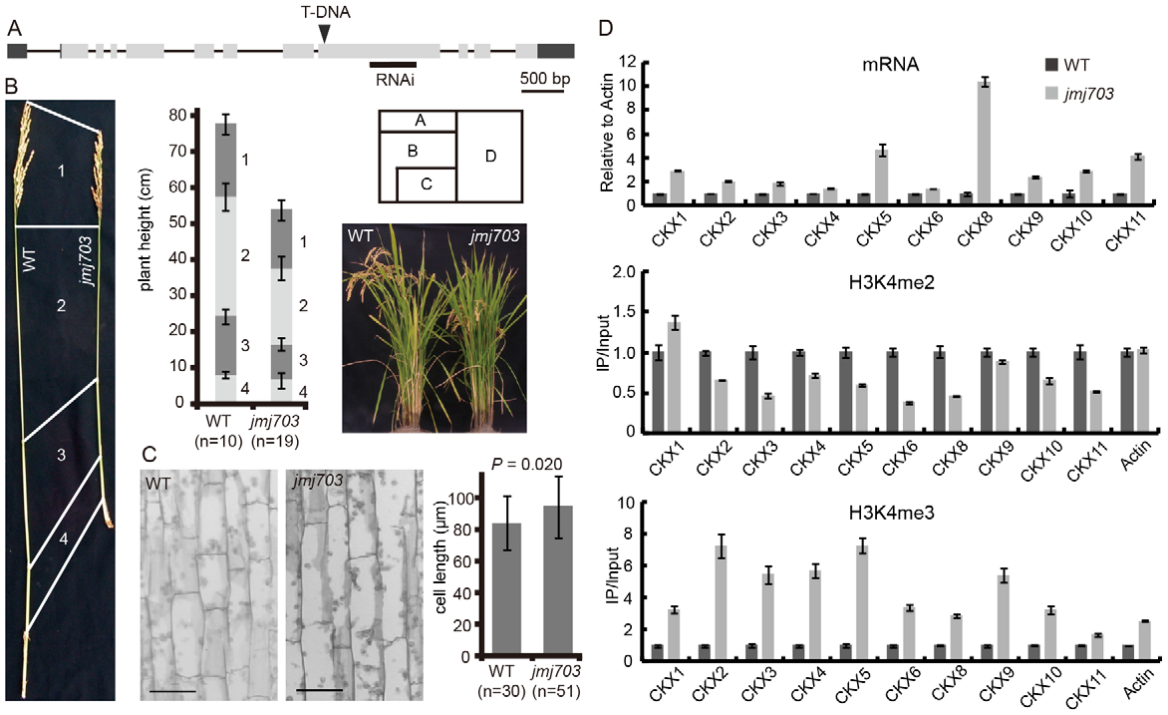
Phenotype of *jmj703* mutants. (A) T-DNA insertion and RNAi locations within the *JMJ703* gene. (B) Comparison of the main panicle internodes between wild type and mutant plants. Lengths of the uppermost internodes were measured. (C) Comparison of stem epidermal cell lengths between wild type and mutant plants. No significant difference of cell lengths between wild type and mutant plants is detected by t-tests (*p* = 0.02). Bar = 50 µm. (D) Relative expression, H3K4me2 and H3K4me3 levels of cytokinin oxidase (*CKX*) genes in wild type and mutant plants. Expression levels in wild type are set as 1.

To investigate the developmental function of *JMJ703*, we characterized a T-DNA insertion mutant and generated several RNAi lines of the gene (Figure 1A). Both knockout and knockdown plants were semi-dwarf and produced smaller seeds (Figure 1B, Figure S1). The phenotypes co-segregated with the T-DNA insertion or the transgene (Table S1). Histological study of stem epidermal tissues revealed no difference in cell length between wild type and the mutant (Figure 1C), suggesting that the shorter stem phenotype may be caused by a slower cell division rate in the *jmj703* mutant plants. Quantitative RT-PCR analysis revealed that the expression of several cell cycle-related genes was unaffected by the mutation (not shown). However, analysis of the cytokinin oxidase (CKX) gene family that reduces active cytokinin levels revealed that several members were highly induced in the young stem of mutant plants (Figure 1D). Chromatin immunoprecipitation assays revealed that H3K4me3 was clearly increased over the promoter region of the *CKX* genes in the mutants (Figure 1D). These data suggested that JMJ703 might regulate H3K4me3 on *CKX* genes and that the mutant phenotype might be due to cytokinin deficiency caused by increased H3K4me3 and increased expression of *CKX* genes.

### JMJ703 is a histone H3K4 demethylase

**Figure 2:**
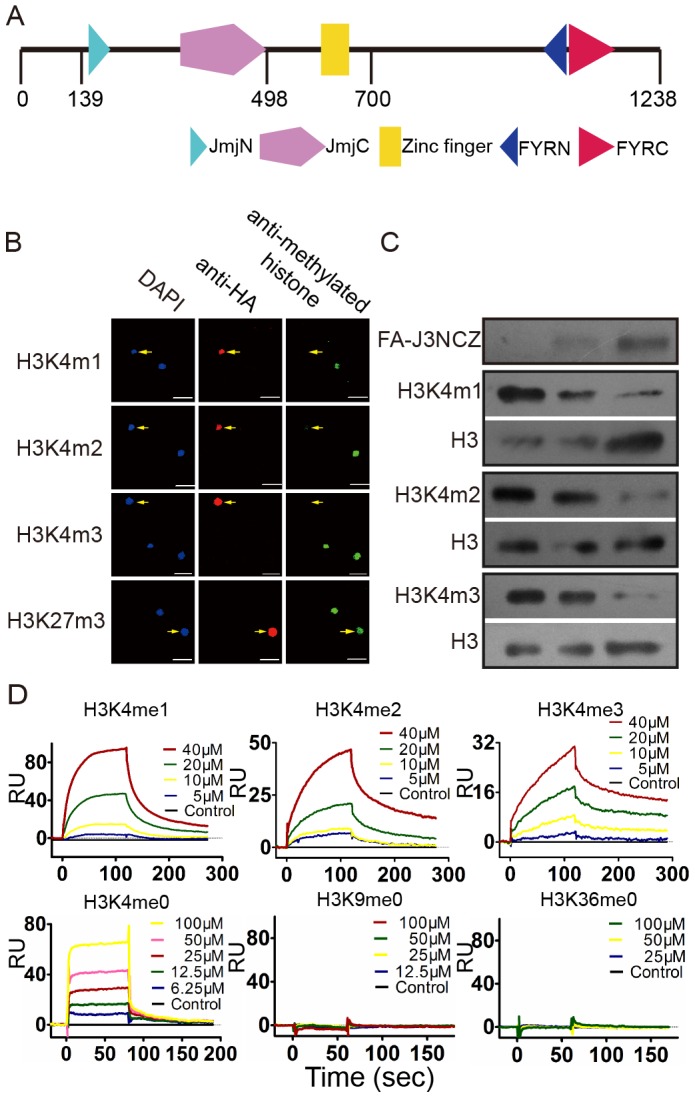
JMJ703 is a histone H3K4 demethylase. (A) Schematic presentation of JMJ703 protein structure. Fragment spanning amino acids 139–498 was obtained for crystal growth and structural determination. The region between amino acids 113–700 was used for enzymatic assays. (B) *In vivo* histone H3K4 demethylase activity of JMJ703. Constructs containing the JMJ703 fragment tagged by FLAG-HA (FA-J3NCZ) were transfected into tobacco leaf cells. Nuclei expressing FA-J3NCZ (stained with anti-HA) were examined for H3K4 methylation by anti-H3K4me1/2/3 and anti-H3K27me3 (indicated by arrows). At least 30 nuclei expressing JMJ703 per transfection were observed and imaged. Bar = 25 µm. (C) *In vitro* demethylase activity of JMJ703. Bulk histones were incubated with three quantities of FA-J3NCZ and analyzed by Western blots using antibodies of H3K4me1/2/3. The same blots were analyzed by anti-H3 to control loadings. FA-J3NCZ levels were revealed by anti-HA. (D) Characterization of c-JMJ703 binding to H3K4me0/1/2/3, H3K9me0 and H3K36me0 peptides using surface plasmon resonance. Curves for different concentration of peptide are differentially colored and labeled.

JMJ703 protein contains several modules, including JmjN, JmjC, C5HC2 zinc finger, FYRN, and FYRC (Figure 2A). The JmjC domain is critical for histone demethylase activity [3]. To investigate the substrate specificity of JMJ703, the FLAG:HA-tagged JmjN-JmjC-zinc finger region (between amino acids 113 and 700, called FA-J3NCZ) of the protein was transiently over-expressed in tobacco leaves for *in vivo* histone demethylase assays. As shown in Figure 2B, nuclei expressing FA-J3NCZ showed a clear decrease of H3K4me1/2/3 compared to non-transfected nuclei, whereas no difference was observed for H3K27me3. FA-J3NCZ was then affinity-purified from transfected tobacco cells for *in vitro* histone demethylase assays. Consistent with the *in vivo* results, FA-J3NCZ could demethylate H3K4me1/2/3 *in vitro* (Figure 2C). By contrast, no activity of FA-J3NCZ to demethylate H3K9me1/2/3, or H3K36me1/2/3 was detected either in tobacco cells or *in vitro* (Figure S2A–S2B), indicating that JMJ703 is an H3K4-specific demethylase.

Although the structures of a number of animal and yeast JmjC proteins have been defined [19]–[21], the structure of an H3K4 demethylase has not yet been reported. To study the structure of JMJ703, we made many attempts, including N- and C-terminal truncations and limited proteolysis, to obtain JMJ703 crystals. Finally, the fragment spanning amino acids 139 to 498 (termed c-JMJ703), covering the JmjN and JmjC domains, was deemed to be suitable for crystallographic investigation. We first performed surface plasmon resonance (SPR) experiments to measure the binding affinities of c-JMJ703 (J3NC) to H3K4 peptides with mono-, di-, or trimethylation. SPR results indicated that c-JMJ703 (J3NC) bound to H3K4me1, H3K4me2, and H3K4me3 peptides with dissociation constant *K_d_* values of 28.9 µM, 19.3 µM, and 30.1 µM, respectively (Figure 2D). In addition, c-JMJ703 (J3NC) could also bind to H3K4me0 peptide, but with a much lower *K_d_* value (76.6 µM), while no binding activity to H3K9me0 and H3K36me0 peptide was observed (Figure 2D). Meanwhile, the fragment corresponding to the JmjN-JmjC-zinc finger region (J3NCZ) showed a higher affinity to H3K4me1/2/3 peptides with a *K_d_* value of 12.6 µM, 15.1 µM and 15.9 µM, respectively (Figure S2C). However, neither c-JMJ703 nor J3NCZ bound to H3K9me3 peptide, suggesting that the zinc finger enhances the substrate binding affinity of JMJ703 but is not essential for the binding specificity (Figure S2D).

### Crystal structure features of c-JMJ703

The crystal structure of c-JMJ703 alone or in complex with α-KG (termed as c-JMJ703-α-KG) or with NOG (N-oxalylglycine, a non-catalytic analog of α-KG) and H3K4me3 peptide (termed as c-JMJ703-NOG-H3K4me3) was determined via the molecular replacement method with the modified crystal structure of the core of human JMJD2A (PDB code 2OQ6) [22] as the initial searching model. The final model was best at a resolution of 2.35 Å with a final *R_work_* value of 19.3% (*R_free_* = 22.5%). The crystals belonged to the *P6_3_* space group with a slight change in unit cell parameters among the three crystals. One c-JMJ703 molecule was identified in the asymmetric unit with a Matthews coefficient of 2.3 Å^3^/Da (corresponding to 46% solvent content) [23]. There are five solvent-exposed regions in c-JMJ703 structure, including P195-K199, S224-R261, R288-S295, T329-Y349, and Q363-V377, which could not be built due to lack of interpretable electron density, suggesting their intrinsic structural flexibility. Moreover, although inter-molecular interactions were found in the c-JMJ703 crystal, gel filtration revealed that c-JMJ703 existed as a monomer in solution (Figure S3), suggesting that the monomer may be the biological unit.

**Figure 3:**
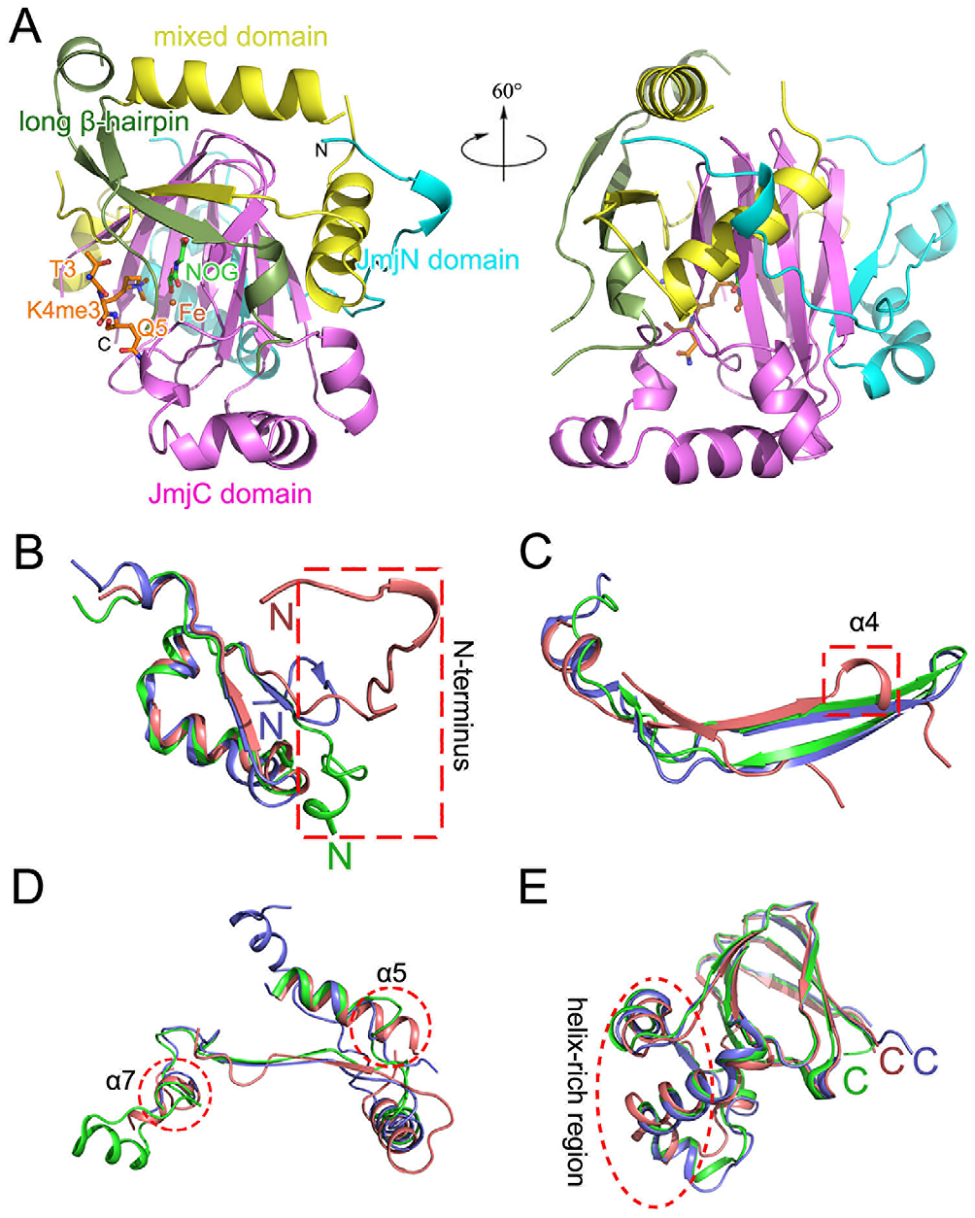
Overall structure of c-JMJ703. (A) Cartoon representation of the overall structure of c-JMJ703. Separate domains are colored differently and labeled. (B–E) Structural comparison of JmjN domain (B), long-beta sheet domain (C), mixed domain (D), and JmjC domain (E) among c-JMJ703 (deep salmon), c-JMJD2A (tv green), and c-Rph1 (slate).

The c-JMJ703 molecule presented a canonical overall folding of JMJD2 proteins and contained four of the five domains defined in the structure of c-JMJD2A: the JmjN domain (A139-K199), the long β-hairpin (D200-T271), the mixed domain (L272-V377) and the JmjC domain (L378-A498) (Figure 3A, Figure S4). The JmjC domain, sandwiched by the JmjN and the long β-hairpin with the mixed domain, adopted a jellyroll-like structure with two four-stranded β-sheets as a cupin fold (Figure 3A) [24].

**Figure 4:**
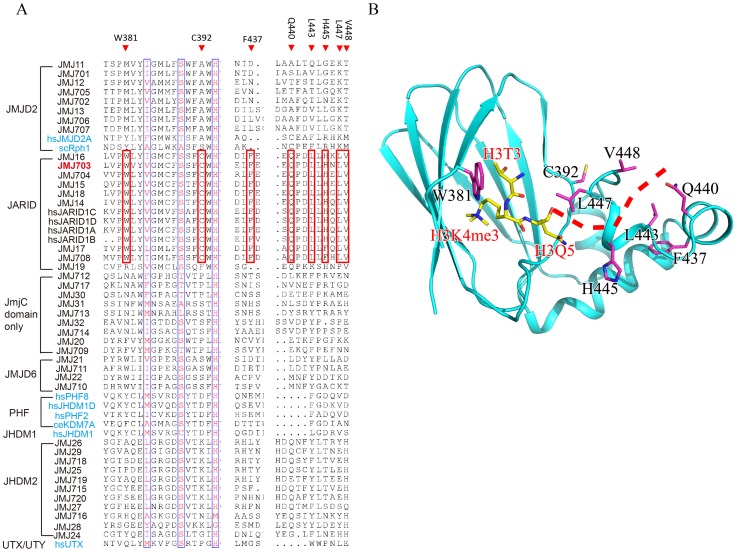
H3K4 demethylase-specific residues within the JmjC domain. (A) Sequence alignment of the JmjC domain of JMJ703, plant JMJ proteins (in black) and representative human/yeast JMJ proteins (in blue). Residues conserved in and specific to H3K4 demethylases are boxed in red and labeled above the alignment. (B) Structure of JmjC domain of JMJ703 showing the locations of H3K4 demethylase-specific residues. H3K4me3 peptide and H3K4 demethylase-specific residues are shown in yellow sticks and purple sticks respectively. A proposed extension path of H3K4me3 peptide toward its C-terminal is shown in dashed red line.

Although c-JMJ703 shared low primary sequence similarity (less than 25% of the sequence identities) with the reported structural homologues [20], [22], the core portion, especially the JmjC domain and the catalytic center of c-JMJ703, presented a topology similar to that of JMJD2 proteins with root mean square derivations of 1.75 Å and 1.81 Å relative to c-JMJD2A and c-Rph1, respectively (Figure 3B–3E). Nevertheless, c-JMJ703 displayed several significant structural differences. First, the JmjN domain presented a number of distinct features (Figure 3B). The orientation of the N-terminus of c-JMJ703 was opposite to that of c-JMJD2A and c-Rph1 (Figure 3B). Second, the long β-hairpin domain contained two β-strands (β2 and β3, aa 209–215 and aa 267–270) and a short α-helix (α4, aa 216–219). The two β-strands of c-JMJ703 were shorter than that of c-JMJD2A and c-Rph1, but had an extra long insertion between them. However, except for the short α-helix (α4), this insertion was mostly invisible in the structure (Figure 3C, Figure S4A). The short α-helix (α4) represented a sharp difference between c-JMJ703 and c-JMJD2A/c-Rph1. Moreover, the mixed domain of c-JMJ703, composed of several different structural elements, also showed clear structural differences. The first α-helix (α5, aa 272–286) of the mixed domain was two turns longer than that of c-JMJD2A and c-Rph1 (Figure 3D). The residues Q363 to V377 in the mixed domain of c-JMJ703 were structurally disordered and presented an uninterpretable electron density, whereas the corresponding region of c-JMJD2A is an α-helix. An additional short helix (α7, aa 356–361) was observed in c-JMJ703, whereas a loop was present in c-JMJD2A (Figure 3D). Several distinct differences could also be observed in the JmjC domain of JMJ703 compared to mammalian and yeast homologues, particularly in the helix-rich region (α8 to α11, aa 419–459) (Figure 3E, Figure S4A). Residues W381, C392, F437, Q440, L443, H445, L447 and V448 are conserved in and specific to JARID and plant H3K4 demethylases (Figure 4A). Most of these residues were located in the helix-rich region (α8 to α11) except W381 and C392 (Figure 4B). A potential extension path of H3K4me peptide toward its C-terminal was proposed based on the locations of these H3K4 demethylase-specific residues (Figure 4B). Substitution mutations of W381 and L447 abolished the H3K4 demethylase activities (Table 1, Figure S6), indicating that these residues are essential for the demethylation function of the protein.

**Table 1:** Summary of H3K4 demethylation assays in tobacco cells of substitution mutants of key residues of JMJ703 catalytic domain.

Function	Residue	H3K4me1	H3K4me2	H3K4me3
Methyl group-binding	G376A	no	no	no
	Y383A	no	yes	no
	E396A	no	no	no
	N496A	no	yes	yes
α-Ketoglutaric acid-binding	Y321A	no	yes	yes
	N404A	yes	yes	yes
	K412A	no	no	no
Fe-binding	H394A	no	no	no
	H482Y	no	no	no
Conserved in H3K4 demethylases	W381A	no	no	no
	L447A	no	no	no
Methylation state-specificity	A494S	no	no	yes

At least 30 nuclei that express JMJ703 per transfection were observed.

### Catalytic machinery of c-JMJ703

**Figure 5:**
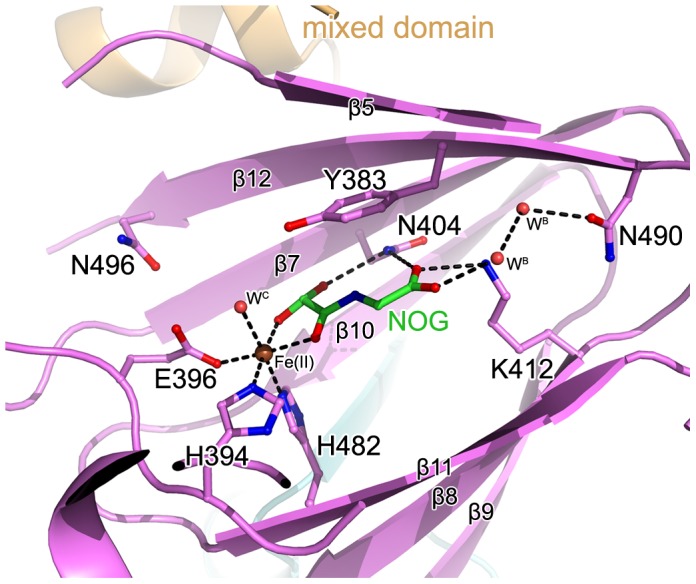
The active site of c-JMJ703. The interaction network of NOG (colored green), Fe(II) (colored brown), and c-JMJ703 residues (colored purple) involved in the interactions are shown. The H3K4me3 peptide is colored yellow. The secondary structure surrounding the active site is shown as a purple cartoon.

The unambiguous electron density denoted Fe(II) (determined by inductively coupled plasma mass spectrometry) bound to c-JMJ703 and identified the active site within the JmjC domain (Figure 5). Three key residues, H394, E396, and H482, are perfectly conserved in JMJD2 proteins. They chelated Fe(II) in the active site through their hydrophilic side chains. Fe(II) also interacted with the C-1 carboxyl and C-2 oxo groups of NOG (Figure 5). Substitution mutations H394A, E396A, and H482A abolished the activity of JMJ703 to demethylate H3K4me1/2/3 (Table 1, Figure S6), confirming their critical role in the enzymatic activity of the protein.

**Figure 6:**
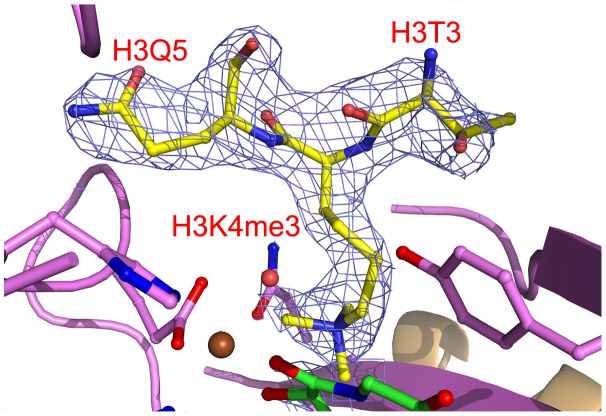
Electron density of bound H3K4me3. The H3K4me3 residues are shown in yellow sticks and labeled in red. Residues surrounding the peptide binding pocket are shown as cartoon and sticks and are colored as in Figure 3. The H3K4me3 peptide is covered by electron density (2Fo-Fc map at 1.0σ).

**Figure 7:**
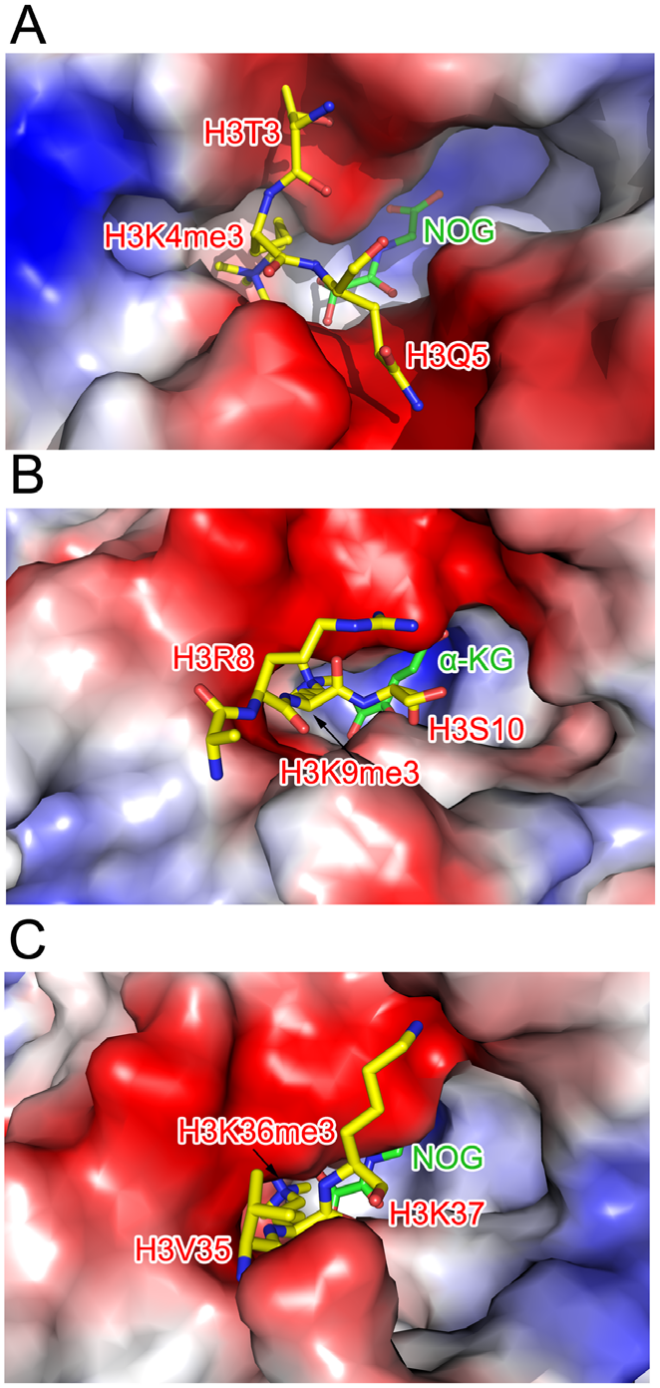
Comparison of the substrate peptide binding modes of c-JMJ703 and c-JMJD2A. (A) Binding of H3K4me3 peptide by c-JMJ703. (B) Binding of H3K9me3 by c-JMJD2A. (C) Binding of H3K36me3 by c-JMJD2A. c-JMJ703 and c-JMJD2A are shown as electrostatic surface and the substrate peptides are shown as yellow sticks and the residues are labeled in red. NOG and α-KG are shown as green sticks and labeled.

The electron density indicated the presence of substrate peptide in the c-JMJ703-NOG-H3K4me3 complex (Figure 3, Figure 6). Three of the ten residues in the H3K4me3 peptide (ARTKme3QTARKS) were visible in the c-JMJ703-NOG-H3K4me3 complex structure (Figure 3, Figure 5, Figure 6). This complex structure provided a view of the substrate peptide-binding mode of an H3K4 demethylase. Compared with the structure of c-JMJD2A in complex with methylated H3K9/H3K36 peptide, the methylated H3K4 and its two flanking residues adopted a different binding conformation to c-JMJ703. H3K9me3/H3K36me3 and their two flanking residues stretched along the long axis of α-KG in c-JMJD2A-α-KG-H3K9me3/c-JMJD2A-NOG-H3K36me3 structure (PDB code 2Q8C/2Q8E, Figure 7B–7C), whereas H3K4me3 and its two flanking residues stretched along the short axis of NOG and was almost perpendicular to H3K9me3/H3K36me3 and their flanking residues (Figure 7A).

The methyl group binding pocket of JmjC domain is unique among methylated peptide- binding proteins due to the polar rather than hydrophobic environment [25]. The methyl groups could not be defined properly in the c-JMJ703-NOG-H3K4me3 complex structure because of low occupancy as indicated by obviously higher B-factor for atoms in H3K4me3 peptide. Based on the crystal structure of c-JMJD2A in complex with H3K9me3 (PDB code 2Q8C) [26], four of the five residues that have been shown to be important in methyl group binding in c-JMJD2A [25] were conserved in c-JMJ703, namely, G376, Y383, E396, and N496. S288 in c-JMJD2A (S335 in Rph1) is substituted by an alanine (A494) in c-JMJ703. G376 had no electron density and was invisible in structure, whereas Y383, E396, A494, and N496 were well-defined and adopted very similar conformations as in c-JMJD2A [26]. Substitution mutations of these potential methyl group-binding residues (G376A, Y383A, E396A, N496A) generally impaired the H3K4 demethylase activity of JMJ703 in tobacco cells, with the exceptions of Y383A, which retained a residual activity to demethylate H3K4me2, and N496A, which was still active to demethylate H3K4me2/3 (Table 1, Figure S6).

### Distinct conformations of Y321 and N404 in stabilizing α-KG

**Figure 8:**
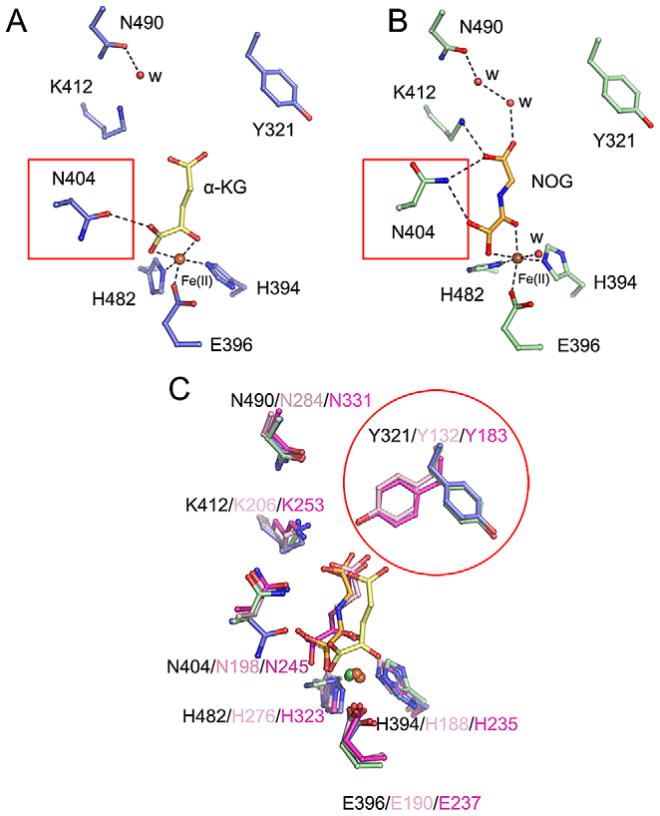
Conformational shifts of α-KG/NOG interacting residues of c-JMJ703 and other members of JMJD2A. Residues interacting with (A) α-KG and (B) NOG in c-JMJ703 structures are represented as blue and green sticks, respectively. Dashed lines denote hydrogen bonds, including the solvent-mediated interactions formed by residues of c-JMJ703 and solvent molecules and bound α-KG/NOG. Fe(II) and solvent molecules are shown as deep red and red spheres, respectively. (C) Comparison of residues involved in binding to Fe(II) and α-KG/NOG in c-JMJ703, c-JMJD2A, and c-Rph1. Residues of c-JMJ703-α-KG, c-JMJ703-NOG-H3K4me3, c-JMJD2A, and c-Rph1 are colored in blue, green, light pink, and purple, respectively. Numbers for each residue in c-JMJ703, c-JMJD2A, and c-Rph1 are shown with black, light pink, and purple, respectively. Solvent molecules are not included in this panel. N404 and Y321, which adopt significant conformation shifts, are framed out by red squares and a circle.

In the crystal structure of c-JMJ703-NOG-H3K4me3, NOG formed three hydrogen bonds with the side chains of N404 and K412 (Figure 5) similar to the JMJD2 homologues. K412A mutation also abolished demethylation activity of H3K4 in all three methylation states (Table 1, Figure S6). Unexpectedly, the N404A mutation produced no clear effect on the demethylase activity of JMJ703, whereas the mutation of the counterpart in Rph1 abolished the H3K36me3 demethylase activity [20]. Moreover, NOG interacted indirectly with N490 through two water molecules (Figure 5), a finding that is not observed in mammalian and yeast homologues. Although most of the residues in α-KG/NOG stabilization showed high structural similarities with its homologues, several features, particularly those of Y321 and N404, distinguished JMJ703 from the JMJD2 members. Y321 and N404 showed significant conformational shifts compared with their counterparts in c-Rph1 and c-JMJD2A (Figure 8A–8C and Figure S5). Alternative conformation of the Tyr corresponding to Y321 in other JmjC proteins has been observed in JMJD2D (PDB code 3DXU). Interestingly, substitution of Y321 by alanine decreased H3K4me1 demethylase activity but did not affect that of H3K4me2 and H3K4me3 (Table 1, Figure S6), whereas substitution by alanine of the corresponding residue in Rph1 leads to loss of the H3K36me3 demethylase activity of the protein [20]. These data suggested a different NOG-binding mechanism between JMJ703 and Rph1.

## Discussion

In this work, we have provided evidence that JMJ703 is a histone demethylase specifically reversing the three forms of methylated H3K4. *Arabidopsis* JMJ14 that belongs to the same group as JMJ703 also has an H3K4 demethylase activity [27]. About two-thirds of plant genes contain at least one type of H3K4me, among which H3K4me3 is associated with gene activation in *Arabidopsis* and rice [28], [29]. Demethylation of the three forms of H3K4me implies that JMJ703 is important for rice gene expression. The phenotype caused by T-DNA insertion and RNAi and increased expression of *CKX* genes suggest that JMJ703 may regulate growth hormone metabolism in rice, which is different from JMJ14 that has functions in floral transition, DNA methylation, and RNA silencing in *Arabidopsis* [27], [30]–[33]. H3K4me3 on specific plant genes can be induced by external signals, such as light, drought, and submergence, among others [34]–[36]. H3K4me3 is suggested to be a mark of active genes and may play a role in plant adaptation to environmental cues. Whether JMJ703 is involved in resetting stress-induced gene expression requires further analysis.

Although JmjC domain of JMJ703 is closely related to that of the JARID proteins [17], the domain organization of the catalytic core of the enzyme (JmjN- long β-hairpin-mixed domain-JmjC) resembles that of JMJD2 members (c-JMJD2A and c-Rph1). However, our functional analysis has revealed that JMJ703 (i.e. FA-J3NCZ) was unable to demethylate H3K9me1/2/3 or H3K36me1/2/3 (Figure S2A–S2B). This suggests that the enzymatic specificity may be determined essentially in the JmjC domain. Our data indicate that the helix-rich region (α8–α11, aa 419–459) of the JmjC domain of JMJ703 is substantially divergent from that of JMJD2A and Rph1. For instance, K241 of c-JMJD2A, which is conserved in c-Rph1, is considered to be a key catalytic residue that recruits and positions O_2_ between Fe(II) and the methyl group to participate in the reaction [25]. Substitution mutation of K241L abolishes the activity of c-JMJD2A [25]. However, this position is replaced by a leucine in JMJ703 (L447) and in other plant homologues and JARID proteins (Figure 4). L447 was found to be essential for the H3K4 demethylase activity of JMJ703 (Table 1, Figure S6). This leucine together with the other residues that are specifically conserved in JARID/H3K4 demethylases and mostly found in the helix-rich region (α8–α11, aa 419–459) of c-JMJ703 (Table 1, Figure 4 and Figure S6), may be involved in the specificity of H3K4 demethylases.

Our data indicate that although most of the JMJ703 residues in α-KG/NOG stabilization showed a high structural similarity with JMJD2 proteins, the residues Y321 and N404 displayed specific features (Figure 8, Figure S5). The conformation of Y321 in c-JMJ703 indicates that the residue may not be in direct contact with α-KG or NOG. Substitution mutation of Y321 only affected H3K4me1 demethylase activity. In addition, N404A substitution did not clearly alter the H3K4 demethylase activity.

The methyl group binding pocket in c-JMJ703 differs also from that of c-JMJD2A and Rph1 in one key residue (A494 in JMJ703 compared to S288 in JMJD2A or S355 in Rph1). S288 of JMJD2A has been shown to be important to reverse di- and trimethylated H3K9 [25], [26], [37]. However, JMJD2D, which harbors an alanine in this position, is 7-fold and 60-fold more efficient than JMJD2A in the demethylation of H3K9me3 and H3K9me2, respectively. JMJD2D has residual activity to H3K9me1 that is absent from JMJD2A [26]. The presence of alanine at this position in JMJ703 (A494) supports the demethylation function of the protein of all three forms of methylated H3K4 (Figure 2). Substitution of this alanine by a serine abolished the demethylase activity of H3K4me1/2, but retained the activity toward H3K4me3 (Table 1, Figure S6). Interestingly, this alanine residue is conserved in most of the plant JMJD2 members (Figure S4), suggesting that these plant proteins may be able to demethylate the three methylated forms of relevant histone lysines.

In addition, the zinc finger that overlaps partially with the JmjC domain of JMJD2A is not found in JMJ703. Although the residues involved in the zinc finger formation in JMJD2A are conserved in Rph1, no zinc ion is observed in its structure and this domain is disordered and invisible in the c-Rph1 structure [20]. By contrast, JMJ703 contains a distal zinc finger motif relative to the JmjC domain (about 100 amino acids downstream the JmjC domain), which is present in Rph1, but absent from JMJD2A. The distal zinc finger is found in a number of other JmjC proteins and is shown to be involved in nuclear localization of JMJ706 [17]. These observations together with our data showing that the inclusion of the motif increased the substrate peptide (H3K4me1/2/3) binding activity to two-folds but not alter the binding specificity, suggest that this zinc finger may not be a component of the catalytic core of JmjC proteins, but is important for their enzymatic activity. Our data also revealed a number of other clear structural differences in the plant JmjC protein. The N-terminal end of c-JMJ703 adopted a completely different conformation from that of c-JMJD2A and c-Rph1. The long β-hairpin domain and mixed domain of c-JMJ703 also showed significant divergence at the amino acid sequence level compared to c-JMJD2A and c-Rph1 (Figure S4). However, the divergent regions are mostly invisible in c-JMJ703 and c-Rph1, indicating intrinsic disorder.

In summary, we have shown that JMJ703 is an H3K4 demethylase that is important for rice plant development. Although JMJ703 displays an overall structure similar to that of JMJD2 H3K9 and H3K36 demethylases, it exhibits a number of important structural features which are conserved within JARID and plant H3K4 demethylases. In particular, our data suggest that the helix-rich region (α8–α11, aa 419–459) of the catalytic JmjC domain of JMJ703 may be involved in the determination of substrate specificity of the H3K4 demethylase.

## Materials and Methods

### T-DNA insertion identification of *jmj703* mutant

A T-DNA insertion line of *JMJ703* (3A-00550) was obtained from the Postech rice mutant database (http://www.postech.ac.kr/life/pfg/risd/). Insertion was confirmed by PCR using the primers muJ3-F and muJ3-R and a T-DNA-specific primer 2715L1 (Table S2).

### RNAi vector construction and rice transformation

A cDNA fragment between nucleotides 2282 bp and 2834 bp relative to the translation start site was amplified using primers RiJ3-F and RiJ3-R and then cloned into T vector (Promega). The fragment was cloned into double strand RNAi vector pDS1301 [38]. Rice transformation with callus generated from the rice cultivar Zhonghua11 (ZH11) was performed as previously described [38].

### Histone demethylation assay

The cDNA fragment of the J3NCZ region was amplified using primers FAJ3NCZ-F and FAJ3NCZ-R and then cloned into pFA121 vector modified from pBI121 (GenBank: AF485783.1), in which GUS was replaced by 2×FLAG∶2×HA tag. The pFA121-J3NCZ plasmid was transferred into *Agrobacterium tumefaciens* strain EHA105 cells for tobacco infection with the help of *Agrobacterium* cells harboring P19 plasmids [39]. For *in vivo* histone demethylation assay, tobacco infection and nuclei isolation were performed as previously described [27]. The immunostaining protocol was modified from http://sites.bio.indiana.edu/~pikaardlab. Briefly, the nucleus solution was placed on a poly-lysine coated slide, air dried, and then refixed with 4% formaldehyde in KPBS (1.28 M NaCl, 20 mM KCl, 80 mM Na_2_HPO_4_, 20 mM KH_2_PO_4_, pH7.2) containing 1% Triton X-100 for 20 min. After 3 washes with KPBS-1% Triton, slides were blocked with blocking solution (1% BSA in KPBS-1% Triton) and incubated at 37°C for 30 min. The blocking solution was washed off with KPBS-1% Triton and the slides were incubated with both anti-HA and histone méthylation specific antibodies diluted in blocking solution to 1∶300 at 4°C overnight. After washing with KPBS, slides were blocked and incubated with fluorescent-labeled antibodies at 37°C for 2 h. After washing with KPBS thrice, nuclei were stained with 5 µg/mL 4,6-diamidino-2-phenylindole (DAPI), coated with a drop of VECTASHIELD Mounting Medium (H-1000, Vector Laboratories), and then covered with a coverslip followed by confocal microscope (Leica) detection. *In vitro* histone demethylation assays were performed as previously described [6]. FA-J3NCZ was purified after overexpression with Anti-FLAG M2 magnetic beads according the manufacturer's instruction (Sigma).

Antibodies used in this study: anti-HA (M20003M, Ab-mart), anti-H3K4me1 (ab8895, Abcam), anti-H3K4me2 (04-790, Millipore), anti-H3K4me3 (07-473, Millipore), anti-H3K27me3 (ABE44, Millipore), anti-H3 (ab1791, Abcam), anti-H3K9me1 (ab9045, Abcam), anti-H3K9me2 (07-441, Millipore), anti-H3K9me3 (ab8898, Abcam), H3K36me1 (ab9048, Abcam), anti-H3K36me2 (ab9049, Abcam), anti-H3K36me3 (ab9050, Abcam), Goat Anti-Mouse secondary antibody (A-11029, Invitrogen), and Goat Anti-Rabbit secondary antibody (A-11036, Invitrogen).

### Chromatin immunoprecipitation

Chromatin immunoprecipitation assays were performed as previously described [38]. Briefly, 2 g of 10 day-old rice seedlings were fixed with 1% formaldehyde, then chromatin was extracted and immunoprecipitated with anti-H3K4me3 (ab8580, Abcam) and anti-H3K4me2 (04-790, Millipore) antibodies. After reversing crosslink and protease K treatment, DNA was recovered for realtime PCR analysis with primers listed in Table S2.

### Protein production for crystallization

DNA fragment encoding amino acids 139 to 498 of wild type *JMJ703* was amplified by PCR, ligated into pET-28a expression vector using *Bam*HI and *Xho*I and transformed into *Escherichia coli* Rosetta (DE3) cells. Transformed *E. c*oli cells were cultured at 37°C in LB medium containing 50 mg/L kanamycin until the OD_600 nm_ reached 0.8. The culture was cooled to 16°C and subsequently induced with 0.5 mM isopropyl β-D-1-thiogalactopyranoside. Cells were harvested after overnight induction by centrifugation at 5,000 *g* for 10 min at 4°C. The cell pellets were resuspended in lysis buffer containing 50 mM Tris (pH 8.0), 500 mM NaCl, and 10% (v/v) glycerol and then disrupted by an ultra-high pressure cell disrupter (JNBIO, Guangzhou, China) at low temperature. Cell debris was removed by centrifugation at 25,000 *g* for 30 min at 4°C. The 6×His tagged protein was purified by Ni-NTA affinity chromatography, cleaved with thrombin (Sigma) overnight at 4°C, and eluted with lysis buffer. The purified c-JMJ703 protein was concentrated and further purified by Hitrap Q (GE Healthcare) anion-exchange chromatography using a 0.05 M to 1 M NaCl gradient in 25 mM HEPES (pH 7.5). The target protein was confirmed to have purity over 95% by SDS-PAGE and concentrated to 10 mg/mL before crystal growth or storage.

### Crystallization

Crystallization was performed at 18°C by the hanging-drop vapor-diffusion technique. Crystals were obtained by mixing 1 µL of the protein solution with an equal volume of a reservoir solution; the mixture drop was equilibrated against 500 µL of the reservoir solution. Crystals were obtained with a reservoir solution containing 0.05 M potassium phosphate monobasic, 25% (w/v) polyethylene glycol 8,000 and reached final dimensions of 50×50×100 µm3 within 2 days. Crystals of apo c-JMJ703 were initially obtained but showed poor diffraction at 3.0 Å resolution and poor reproducibility. Crystals of c-JMJ703 in complex with NOG displayed much better diffraction quality at 2.3 Å, indicating that NOG helps stabilize the conformation of the c-JMJ703 polypeptide. Crystals of the c-JMJ703 substrate complex were obtained by co-crystalizing c-JMJ703 with 10 mM peptide. Crystals were cryo-protected by soaking in a cryo-protectant consisting of the reservoir solution with additional 15% (v/v) glycol. Cryo-protected crystals were then flash-cooled in liquid nitrogen and transferred into a dry nitrogen stream at 100 K for X-ray data collection.

### X-Ray data collection, processing, and structure determination

Diffraction data for apo c-JMJ703 crystals were collected at a resolution of 3.0 Å at 100 K using a MAResearch M165 CCD detector in beamline 1W2A at the Beijing Synchrotron Radiation Facility. The data set for c-JMJ703-α-KG and c-JMJ703-NOG-H3K4me3 were collected at 2.3 Å and 2.4 Å in beamline BL17A (Photon Factory, Japan) and BL17U1 (SSRF, Shanghai, China), respectively, with an ADSC Q315 CCD detector at the wavelength of 1.0000 Å. All data sets were indexed, integrated, and scaled using the HKL2000 package [40]. The crystals belonged to space group *P6_3_* with cell parameters *a = *53.0 Å, *b = *86.1 Å, *c = *87.3 Å, and β = 90.7°. All crystals showed very small shifts in their cell parameters.

The molecular replacement method was used to calculate the phases using the PHASER program [41] and the modified crystal structure of a catalytic core of the human c-JMJD2A (PDB code 2OQ6) as the initial searching model. Manual model building and refinement were performed with the programs COOT [42] and PHENIX [43]. Solvent molecules were located from stereochemically reasonable peaks in the σA-weighted 2*Fo–Fc* difference Fourier electron density map (1.2 σ). Model geometry was verified using the program PROCHECK (Table S3) [44]. Coordinates have been deposited in PDB with accession 4IGP, 4IGO and 4IGQ for apo c-JMJ703, c-JMJ703-α-KG and c-JMJ703-NOG-H3K4me3, respectively.

### Surface plasmon resonance binding analysis

Analyses were carried out at 25°C with the BIAcore 3000 system. Ten µg/mL c-JMJ703 in 10 mM sodium acetate buffer (pH 5.0) was covalently coupled to a CM5 chip (Biacore) using an Amine Coupling Kit (Biacore) according to the manufacturer's instructions. Using HEPES buffer (10 mM HEPES pH 7.5, 150 mM NaCl, 0.005% Tween20), various concentrations of the H3K4me1/2/3 peptide were injected through a flow cell that was not activated and then through another flow cell containing c-JMJ703 at a rate of 10 µL/min for 2 min. The c-JMJ703 surface was regenerated between two injections by running 15 µL of 5 mM NaOH twice through the flow cell at 30 µL/min. The sensorgram obtained for the inactivated flow cell was subtracted to correct for nonspecific binding and the bulk signal from the peptide in solution. Data were analyzed using BIAevaluation 4.1 software.

## Supporting Information

Figure S1 Phenotype of *JMJ703* RNAi and mutant plants. (A) *JMJ703* RNAi plants show semi-dwarf phenotype, which is similar to *jmj703* mutant. (B) Smaller seed and panicle enclosure phenotypes. Bar = 0.5cm.
(TIF)

Figure S2 JMJ703 histone demethylation and substrate binding activities. (A) *In vivo* analysis of JMJ703 (FA-J3NCZ) H3K9 and H3K36 demethylation activity in tobacco cells. Bar = 10 µm. (B) *In vitro* assays of JMJ703 H3K9 and H3K36 demethylation activity with tobacco cell expressed FA-J3NCZ. (C) Characterization of binding of FA-J3NCZ to H3K4me1/2/3 peptides using surface plasmon resonance. Curves for different concentration of peptide are differentially colored and labeled. The peptide name is labeled above the curve. (D) Binding assays of J3NC and J3NCZ fragments to H3K9me peptides.
(TIF)

Figure S3 Size exclusion chromatography of c-JMJ703. C-JMJ703 (20 mg/mL) was injected onto a Superdex 75 10/300 GL column with the elution buffer containing 20 mM HEPES pH7.5, 150 mM NaCl. The retention volume is 11.5 mL. Retention volumes for molecular weight standards are shown above.
(TIF)

Figure S4 Key catalytic core sequence comparison between JMJ703, structurally studied JmjC proteins and other plant JmjC proteins. (A). Sequence alignment of c-JMJ703, c-JMJ16, c-JMJD2A and c-Rph1. The secondary structure of c-JMJ703 is shown and labeled above the alignment. The color scheme is the same as Figure 3. Residues involved in Fe(II), α-KG and methyl group binding and previously proposed O_2_-recruiting lysine are shown beneath the alignment in brown triangle, green square, light blue circle and red circle, respectively. (B). Sequence alignment of the rice (Jmj701–720) and Arabidopsis JmjC (Jmj11–32) proteins with representative animal/yeast proteins. JmjC proteins for which the crystal structure has been determined are highlighted by blue. Only key residue regions are shown. Key residues identified in the structure of JMJ703 are indicated by stars at the bottom, with residues involved in Fe(II) binding in purple, α-KG binding in brown, methyl group binding in blue and the previously proposed O_2_-recruiting leucine in yellow.
(TIF)

Figure S5 Electron density of bound α-KG/NOG and interacting residues of c-JMJ703. The residues interact with bound α-KG (A) and NOG (B) in the complex structure of c-JMJ703-α-KG and c-JMJ703-NOG-H3K4me3 are shown as blue and green sticks, respectively. Fe(II) and solvent molecules, which mediate interaction between polypeptide and compounds, are presented by colored spheres. All components are covered by electron density (2Fo-Fc map at 1.1σ).
(TIF)

Figure S6 Demethylation assays for JMJ703 substitution mutants that have been produced based on the structural data. The mutant names are labeled at the top left corner of each panel. Image panels from left to right are staining by DAPI, anti-HA and anti-methylated histones, respectively. Bar = 10 µm. At least 30 nuclei that expressed JMJ703 per transfection were observed and imaged.
(TIF)

Table S1 Internode lengths of JMJ703 RNAi plants and T-DNA mutants.
(DOC)

Table S2 Primers used in this study.
(DOCX)

Table S3 Data collection and refinement statistics.
(DOCX)
