# Supplementary figures and images for: Structural Basis of a Histone H3 Lysine 4 Demethylase Required for Stem Elongation in Rice
Source: PLoS Genet. 2013 Jan 24;9(1):e1003239. doi: 10.1371/journal.pgen.1003239 (PMC3554631; doi:10.1371/journal.pgen.1003239)

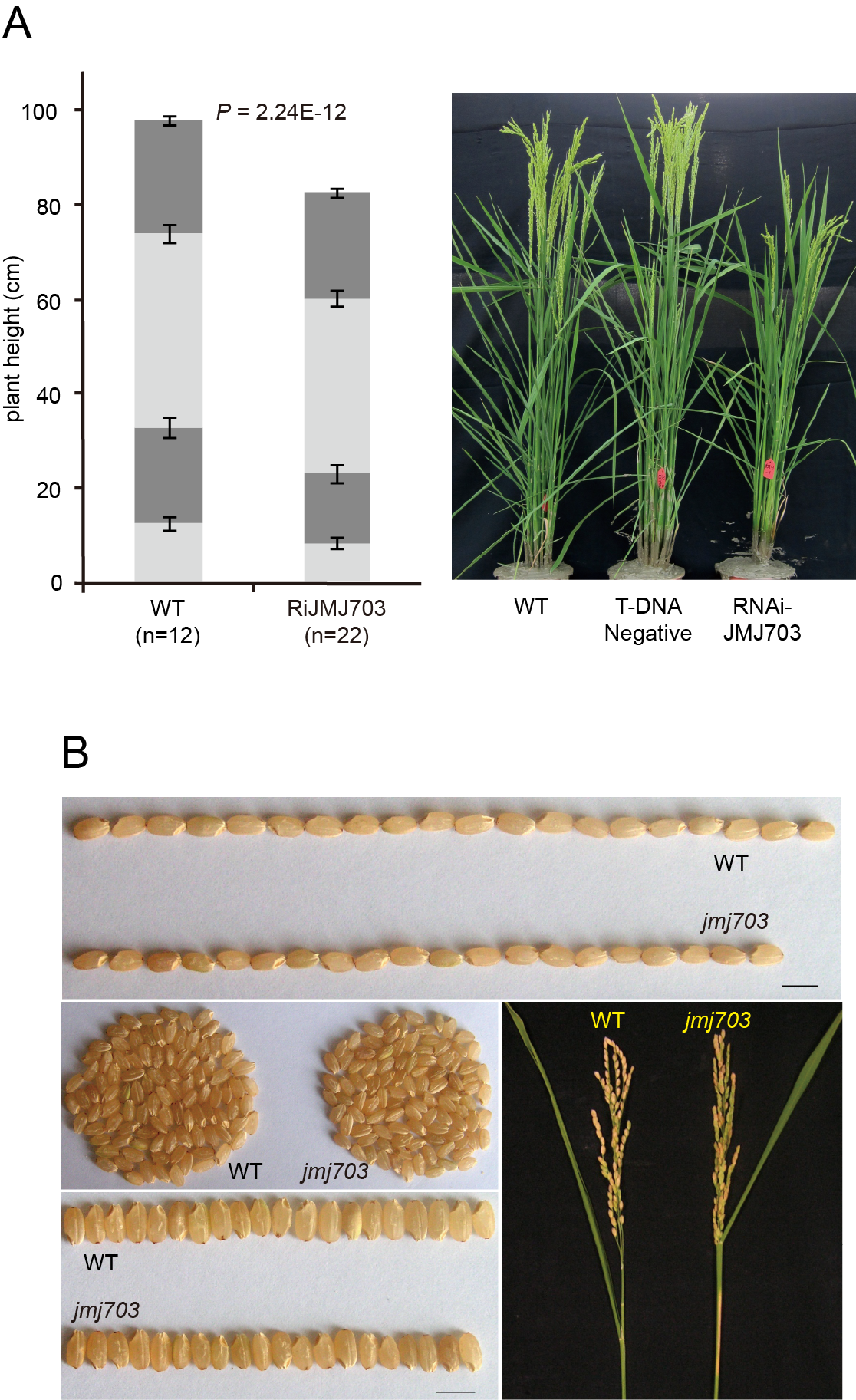

Supplement: Figure S1 — Phenotype of JMJ703 RNAi and mutant plants. (A) JMJ703 RNAi plants show semi-dwarf phenotype, which is similar to jmj703 mutant. (B) Smaller seed and panicle enclosure phenotypes. Bar = 0.5cm. (TIF) [file pgen.1003239.s001.tif]

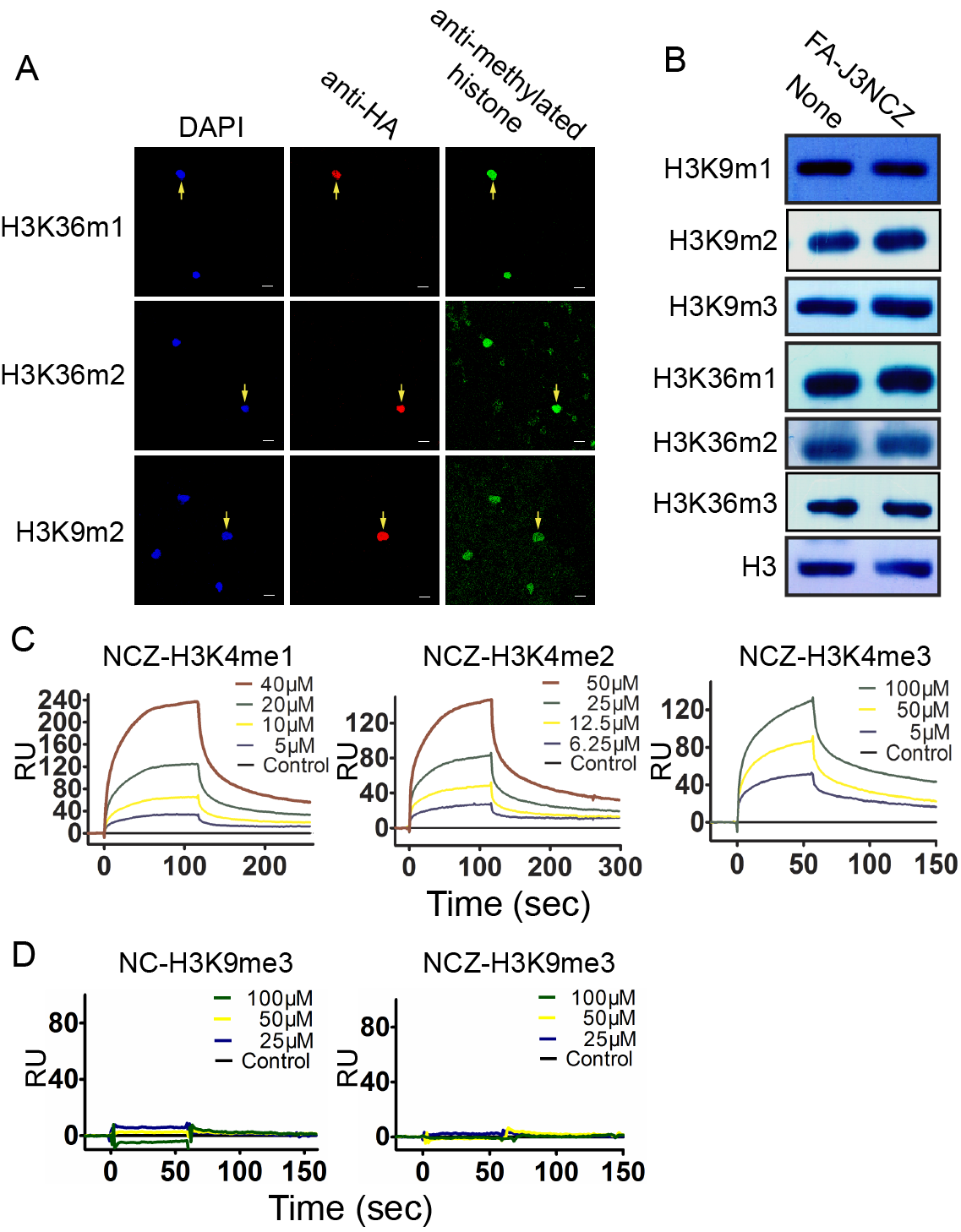

Supplement: Figure S2 — JMJ703 histone demethylation and substrate binding activities. (A) In vivo analysis of JMJ703 (FA-J3NCZ) H3K9 and H3K36 demethylation activity in tobacco cells. Bar = 10 µm. (B) In vitro assays of JMJ703 H3K9 and H3K36 demethylation activity with tobacco cell expressed FA-J3NCZ. (C) Characterization of binding of FA-J3NCZ to H3K4me1/2/3 peptides using surface plasmon resonance. Curves for different concentration of peptide are differentially colored and labeled. The peptide name is labeled above the curve. (D) Binding assays of J3NC and J3NCZ fragments to H3K9me peptides. (TIF) [file pgen.1003239.s002.tif]

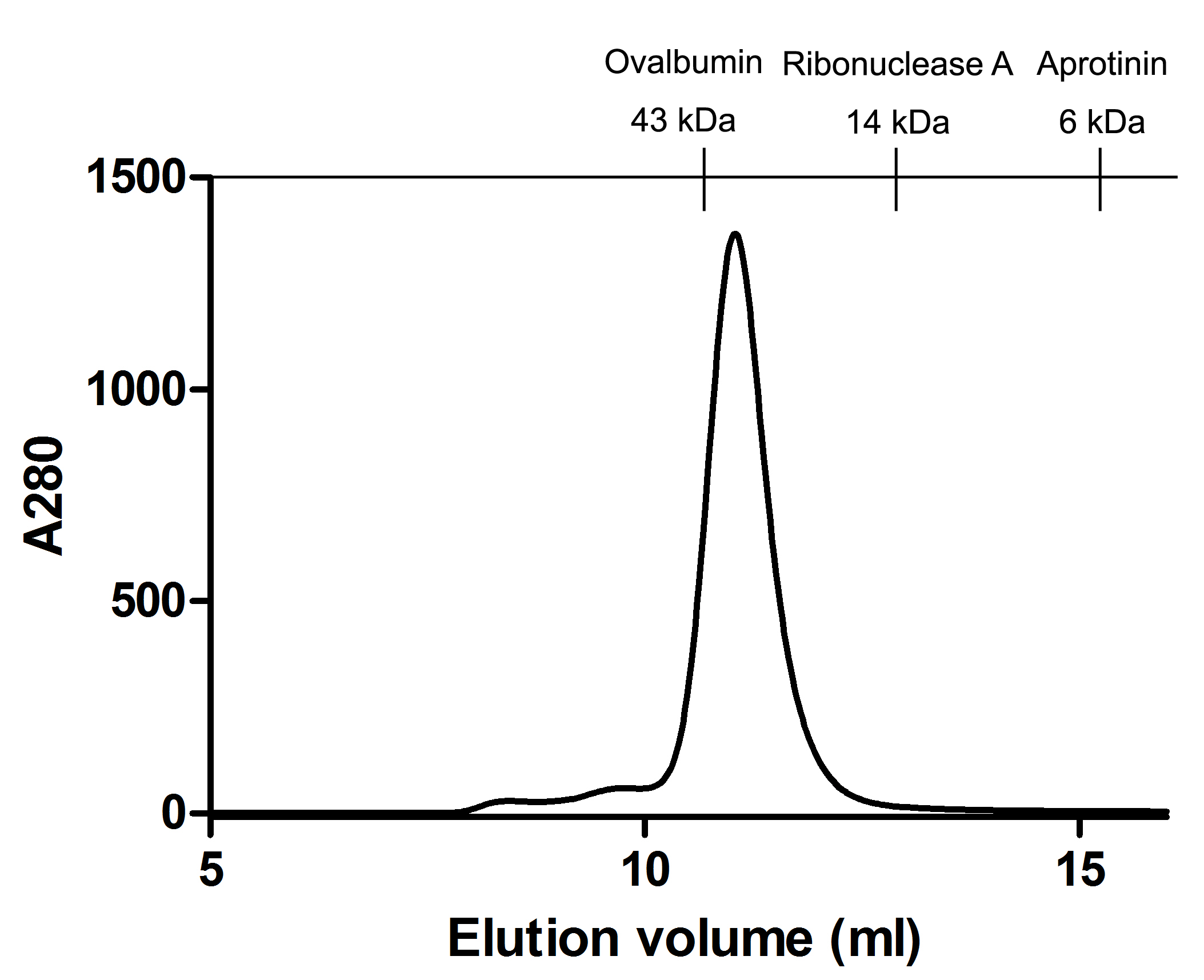

Supplement: Figure S3 — Size exclusion chromatography of c-JMJ703. C-JMJ703 (20 mg/mL) was injected onto a Superdex 75 10/300 GL column with the elution buffer containing 20 mM HEPES pH7.5, 150 mM NaCl. The retention volume is 11.5 mL. Retention volumes for molecular weight standards are shown above. (TIF) [file pgen.1003239.s003.tif]

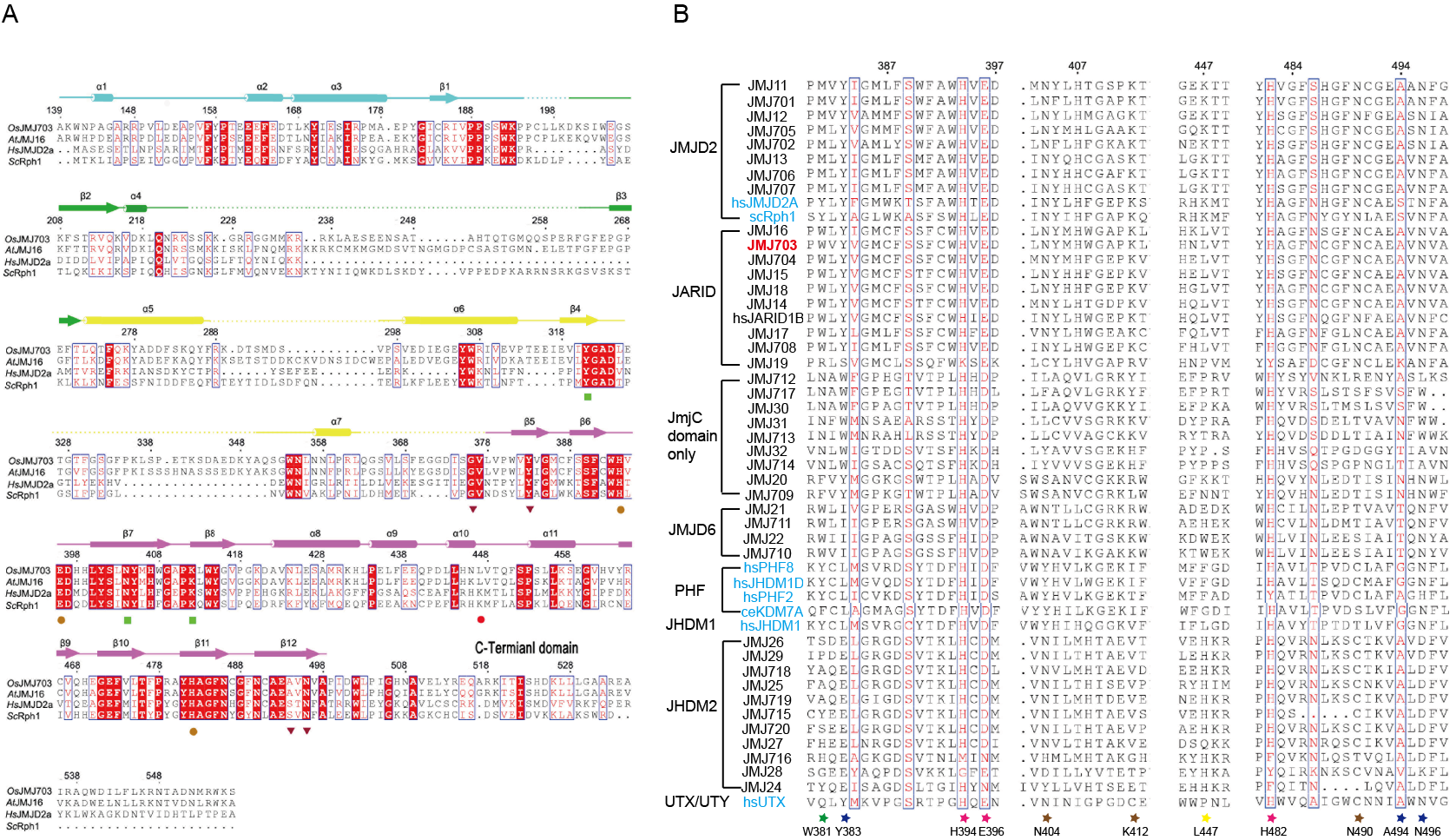

Supplement: Figure S4 — Key catalytic core sequence comparison between JMJ703, structurally studied JmjC proteins and other plant JmjC proteins. (A). Sequence alignment of c-JMJ703, c-JMJ16, c-JMJD2A and c-Rph1. The secondary structure of c-JMJ703 is shown and labeled above the alignment. The color scheme is the same as Figure 3. Residues involved in Fe(II), α-KG and methyl group binding and previously proposed O2-recruiting lysine are shown beneath the alignment in brown triangle, green square, light blue circle and red circle, respectively. (B). Sequence alignment of the rice (Jmj701–720) and Arabidopsis JmjC (Jmj11–32) proteins with representative animal/yeast proteins. JmjC proteins for which the crystal structure has been determined are highlighted by blue. Only key residue regions are shown. Key residues identified in the structure of JMJ703 are indicated by stars at the bottom, with residues involved in Fe(II) binding in purple, α-KG binding in brown, methyl group binding in blue and the previously proposed O2-recruiting leucine in yellow. (TIF) [file pgen.1003239.s004.tif]

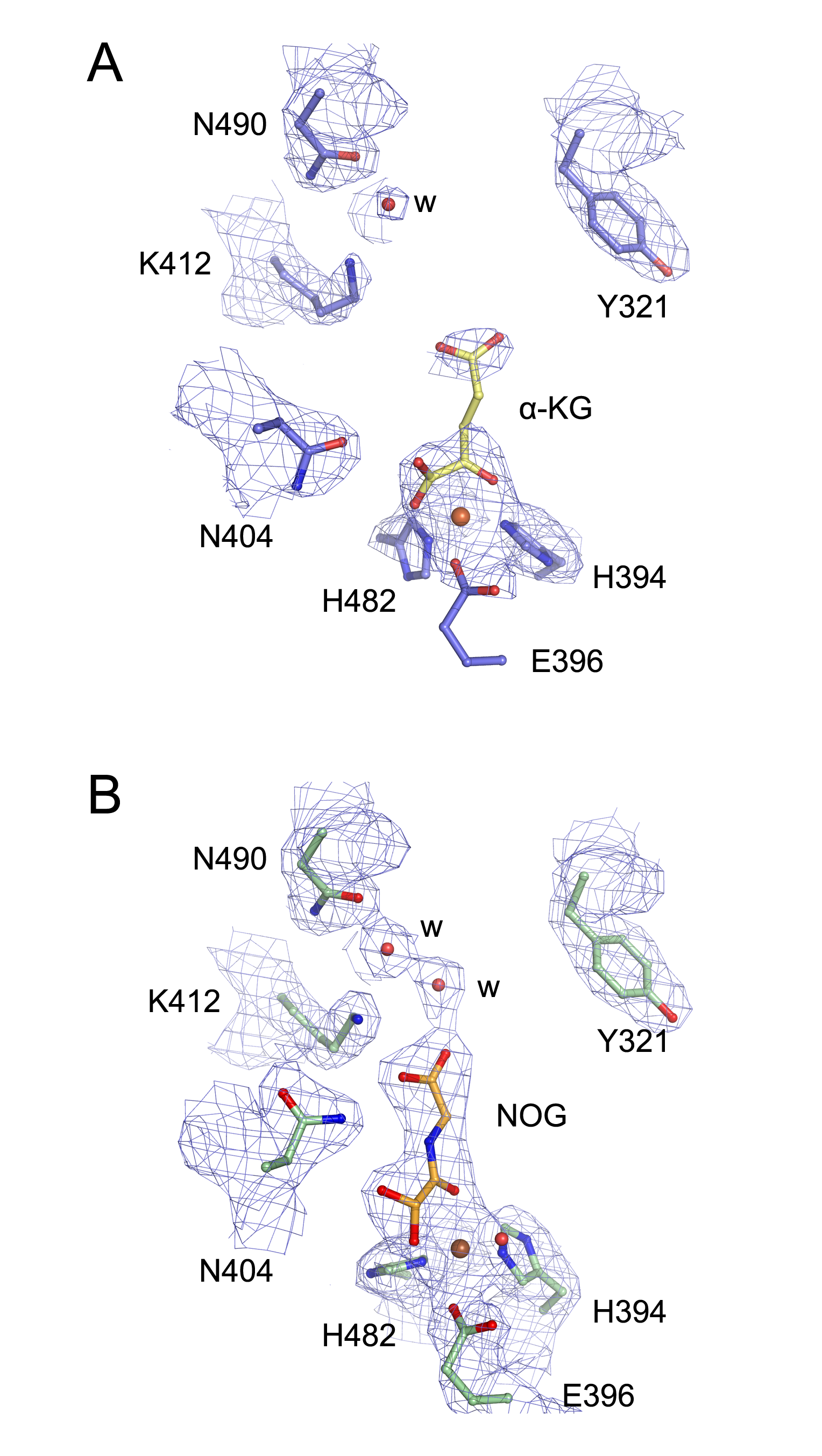

Supplement: Figure S5 — Electron density of bound α-KG/NOG and interacting residues of c-JMJ703. The residues interact with bound α-KG (A) and NOG (B) in the complex structure of c-JMJ703-α-KG and c-JMJ703-NOG-H3K4me3 are shown as blue and green sticks, respectively. Fe(II) and solvent molecules, which mediate interaction between polypeptide and compounds, are presented by colored spheres. All components are covered by electron density (2Fo-Fc map at 1.1σ). (TIF) [file pgen.1003239.s005.tif]

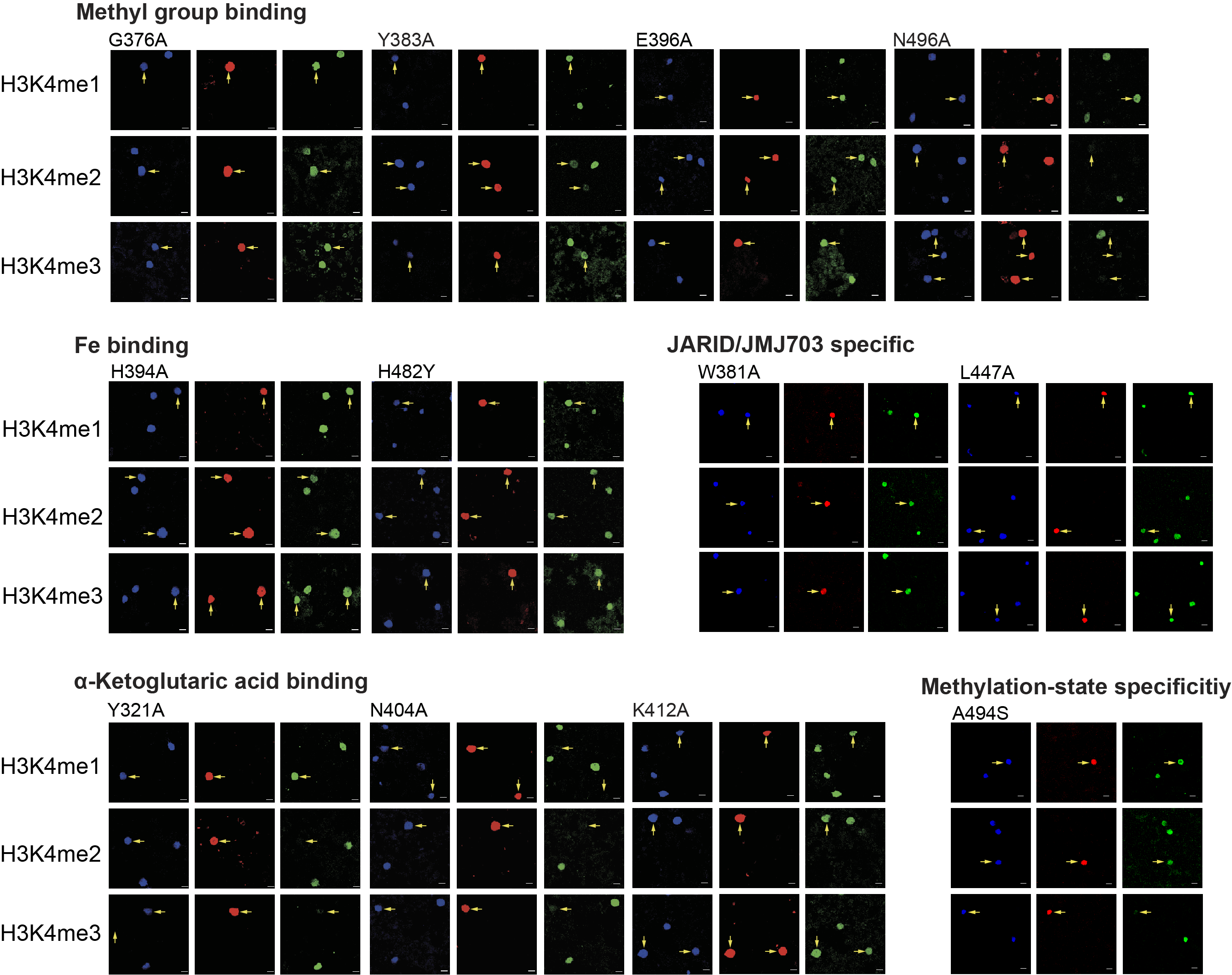

Supplement: Figure S6 — Demethylation assays for JMJ703 substitution mutants that have been produced based on the structural data. The mutant names are labeled at the top left corner of each panel. Image panels from left to right are staining by DAPI, anti-HA and anti-methylated histones, respectively. Bar = 10 µm. At least 30 nuclei that expressed JMJ703 per transfection were observed and imaged. (TIF) [file pgen.1003239.s006.tif]
