# Supplementary material for: Structural Basis of a Histone H3 Lysine 4 Demethylase Required for Stem Elongation in Rice
Source: PLoS Genet. 2013 Jan 24;9(1):e1003239. doi: 10.1371/journal.pgen.1003239 (PMC3554631; doi:10.1371/journal.pgen.1003239)
Supplement: Table S1 — Internode lengths of JMJ703 RNAi plants and T-DNA mutants. (DOC) [file pgen.1003239.s007.doc]

## Table S1. Length of each internode of JMJ703 knockdown and knockout plants

|  | Genotype | NO. of  plants | Length (cm) | | | |
| --- | --- | --- | --- | --- | --- | --- |
|  |  |  | Panicle | Internode1 | Internode2 | Internode3 |
| RNAi | Negative | 12 | 23.75±0.89 | 41.13±1.81 | 20.29±2.18 | 12.33±1.59 |
|  | Positive (T1 from 3 lines) | 22 | 22.18±0.93 | 37.00±1.83 | 14.80±1.86 | 8.20±1.18 |
|  | *t* |  | 4.76 | 6.30 | 7.75 | 8.62 |
|  | *p* |  | 4.04E-05 | 4.58E-07 | 7.81E-09 | 7.5E-10 |
| mutant | Negative | 10 | 20.31±2.89 | 33.33±4.13 | 16.33±2.10 | 7.86±1.12 |
|  | Positive (T2) | 19 | 16.27±3.03 | 21.30±3.35 | 9.88±1.81 | 6.46±2.16 |
|  | *t* |  | 3.46 | 8.48 | 8.64 | 1.90 |
|  | *p* |  | 1.82E-03 | 4.25E-09 | 2.96E-09 | 6.76E-02 |

All data are given as mean ± SD. *P* value were obtained by *t* tests between positive and negative plants.

## 
