# Supplementary material for: Structural Basis of a Histone H3 Lysine 4 Demethylase Required for Stem Elongation in Rice
Source: PLoS Genet. 2013 Jan 24;9(1):e1003239. doi: 10.1371/journal.pgen.1003239 (PMC3554631; doi:10.1371/journal.pgen.1003239)
Supplement: Table S2 — Primers used in this study. (DOCX) [file pgen.1003239.s008.docx]

## Table S2. Primers used in this study

| Primer name | sequence |
| --- | --- |
| muJ3-F | TCTACCGCAGGTTACCCAGTTT |
| muJ3-R | CAGCCAGAAGCAGAAAGATGTAAAT |
| 2715L1 | CGTCCGCAATGTGTTATTAAG |
| RiJ3-F | GGGACTAGTGGTACCGGAATGAGTTGTCATGTACAACAAA |
| RiJ3-R | GGGGAGCTCGGATCCGCAGGGGGATCTAAAGAAGG |
| FAJ3NCZ-F | GCTCTAGAATGACGAGACGCAGACAACAGCT |
| FAJ3NCZ-R | GCTCTAGATGGACCATCAGTTAATCTGCG |
| 139F | CCCCCCGCGGATCCGCAAAATGGAATCCAGCT |
| 498R | CCCCCCCGCTCGAGCTAAGCCACATTAACAGCTTCGG |
| WEE1-F | AAAATTGCGTTGCTTCCAGG |
| WEE1-R | AGGACTTCTTTCGCAGATGGC |
| KRP4-F | ACACCTTGCAGCTTGATCAGG |
| KRP4-R | CAGCTCTGCTGATGCTGGAAT |
| KRP5-F | ACCCCTGGCTCCACAACTAAA |
| KRP5-R | AGCCTTGTCCATTCGTACCGT |
| RBR1-F | CGGCTATCTCGGTTTCCAA |
| RBR1-R | TCTTGAGGCGTACCTTTGATGT |
| RBR2-F | GCCGCCTAAATTACCCTACCA |
| RBR2-R | CGAAGAGGCAGAACCACCATT |
| CKX1 (LOC_Os05g31040) | |
| mRNA-F | TGACAAGGCAGGTATTGGAGTG |
| mRNA-R | TCCTCTGTTGAAATGTGTCCCA |
| ChIP-F | CAGTGCCCAATACCGGTTTG |
| ChIP-R | TGTATGTGGACTGGTGCAGGA |
| CKX2 (LOC_Os01g10110) | |
| mRNA-F | ATCTACCCCATGAACCGCAAC |
| mRNA-R | TGCACGAATCTTGGCCAGA |
| ChIP-F | GGAATCAAACCTAGGGTGGCT |
| ChIP-R | CTTGGGCCTAATGGCTAGCA |
| CKX3 (LOC_Os01g09260) | |
| mRNA-F | CTCATCATCTACCCCGTCAACA |
| mRNA-R | ACTTGTCCTTCCTCTGCACGA |
| ChIP-F | CGTCAAAGAATCCCATTGTGATT |
| ChIP-R | CCAGGAGATTGTTGCCAAAATTA |
| CKX4 (LOC_Os01g71310) | |
| mRNA-F | AGAAGCAGTGGAAAGCCCACTT |
| mRNA-R | TGGAAATATTCTCTGCCCTGGA |
| ChIP-F | TCCCGAACAGTGCTAGGATTG |
| ChIP-R | GGCTCCATCGTCGTAAGTGG |
| CKX5 (LOC_Os06g37500) | |
| mRNA-F | TTCCTTCTTCTCTGCAACCGA |
| mRNA-R | TTTCTGATCCACGGAAGCG |
| ChIP-F | CCACTCAACCAAACACACCCT |
| ChIP-R | TGCCCAACAAACAATTCTTCC |
| CKX6 (LOC_Os06g37500) | |
| mRNA-F | CGTGCAATTCCTTGATCGTG |
| mRNA-R | ACCGGTGTCGAAGTCAAGGAT |
| ChIP-F | CTGATCAACTGCAAAGCGTCC |
| ChIP-R | GCAATCGGATTAGCGAACTGA |
| CKX8 (LOC_Os01g56810) | |
| mRNA-F | AGAATTACGACGACTCCACCGC |
| mRNA-R | TCTAGGAAGTCCACGTACGGCA |
| ChIP-F | CGAGCAAATACATCGCCATCT |
| ChIP-R | TTGCGTGCACTATGACGACA |
| CKX9 (LOC_Os08g35860) | |
| mRNA-F | CAACGCAATCATTGACGCC |
| mRNA-R | ATCGAACCGAAACCTCCCTCT |
| ChIP-F | AGGTAGCTAACCATTCGCGC |
| ChIP-R | TCGGATCTAGCGTTTGCTTGT |
| CKX10 (LOC_Os04g44230) | |
| mRNA-F | AATGTGTTCGTGCCAAAGCA |
| mRNA-R | ACGTGTTCCCGTCCCACTTAT |
| ChIP-F | ACGTTTCATGACAGGCTCTCG |
| ChIP-R | GCCTCTGTCCTGACGGTCAA |
| CKX11 (LOC_Os10g34230) | |
| mRNA-F | AACATGTTCGTGCCAAGCG |
| mRNA-R | ACGTGTTGGTGTCCCACTTGT |
| ChIP-F | CCTCTTTTTGGTACAGTCTTCGGA |
| ChIP-R | GATCACACTGCACACACTCTCTCC |
