# Supplementary material for: Structural Basis of a Histone H3 Lysine 4 Demethylase Required for Stem Elongation in Rice
Source: PLoS Genet. 2013 Jan 24;9(1):e1003239. doi: 10.1371/journal.pgen.1003239 (PMC3554631; doi:10.1371/journal.pgen.1003239)
Supplement: Table S3 — Data collection and refinement statistics. (DOCX) [file pgen.1003239.s009.docx]

## Table S3. Data collection and refinement statistics.

| Parameters | apo JMJ703 | JMJ703+α-KG | JMJ703+NOG  +H3K4me3 | |
| --- | --- | --- | --- | --- |
| Data collection statistics | | | |  |
| Cell parameters |  |  |  | |
| *a* (Å) | 91.3 | 90.5 | 91.4 | |
| *b* (Å) | 91.3 | 90.5 | 91.4 | |
| *c* (Å) | 76.0 | 76.0 | 75.8 | |
| α, β, γ (°) | 90.0, 90, 120.0 | 90.0, 90, 120.0 | 90.0, 90.0, 120.0 | |
| Space group | *P6_3_* | *P6_3_* | *P6_3_* | |
| Wavelength used (Å) | 1.0000 | 1.0000 | 1.0000 | |
| Resolution (Å) | 50.0 (3.1)^c^ -3.0 | 50.00 (2.48) – 2.40 | 50.00 (2.39) – 2.35 | |
| No. of all reflections | 56,883 (3,293) | 141,165 (10,900) | 123,279 (6,451) | |
| No. of unique reflections | 6,821 (383) | 16,249 (1,617) | 15,151 (759) | |
| Completeness (%) | 93.1 (100.0) | 100.0 (100.0) | 100.0 (100.0) | |
| Average I/σ(I) | 6.1 (4.1) | 40.5 (4.9) | 30.7 (4.7) | |
| R_merge_^a^ (%) | 14.6 (53.2) | 10.6 (53.5) | 12.8 (58.4) | |
| Refinement statistics | | | |  |
| No. of reflections used (σ(F) > 0) | 6,629 | 26,050 | 15,108 | |
| R_work_^b^ (%) | 22.7 | 19.3 | 17.9 | |
| R_free_^b^ (%) | 28.6 | 22.9 | 22.1 | |
| r.m.s.d. bond distance (Å) | 0.011 | 0.009 | 0.009 | |
| r.m.s.d. bond angle (º) | 1.507 | 1.268 | 1.230 | |
| No. of protein atoms | 2,206 | 2,306 | 2,845 | |
| No. of protein atoms | 2,206 | 2,205 | 2,383 | |
| No. of ligand atoms | 0 | 11 | 23 | |
| No. of solvent atoms | 0 | 90 | 142 | |
| B-value (Å^2^) |  |  |  | |
| B-value for protein | 40.9 | 54.8 | 42.0 | |
| B-value for ligand/ion | - | 68.7 | 59.4 | |
| B-value for solvent | - | 52.9 | 42.8 | |
| Ramachandran plot |  |  |  | |
| Res. in favored regions (%) | 80.6 | 87.8 | 90.7 | |
| Res. in additional allowed regions (%) | 19.4 | 12.2 | 9.3 | |
| Res. in disallowed regions (%) | 0 | 0 | 0 | |

^a^*R_merge_* = Σ_h_Σ_l_ | I_ih_−<I_h_> |/Σ_h_Σ_I_ <I_h_>, where <I_h_> is the mean of the observations I_ih_ of reflection h.

^b^*R_work_* = Σ( ||F_p_(obs)|−|F_p_(calc)||)/ Σ|F_p_(obs)|; *R_free_* is an R factor for a selected subset (5%) of the reflections that was not included in prior refinement calculations.

^c^Numbers in parentheses are corresponding values for the highest resolution shell.
